# Plasmid-free CRISPR/Cas9 genome editing in *Plasmodium falciparum* confirms mutations conferring resistance to the dihydroisoquinolone clinical candidate SJ733

**DOI:** 10.1371/journal.pone.0178163

**Published:** 2017-05-22

**Authors:** Emily D. Crawford, Jenai Quan, Jeremy A. Horst, Daniel Ebert, Wesley Wu, Joseph L. DeRisi

**Affiliations:** 1 Chan Zuckerberg Biohub, San Francisco, California, United States of America; 2 Department of Biochemistry and Biophysics, University of California San Francisco, San Francisco, California, United States of America; 3 Howard Hughes Medical Institute, Chevy Chase, Maryland, United States of America; Osaka University, JAPAN

## Abstract

Genetic manipulation of the deadly malaria parasite *Plasmodium falciparum* remains challenging, but the rise of CRISPR/Cas9-based genome editing tools is increasing the feasibility of altering this parasite’s genome in order to study its biology. Of particular interest is the investigation of drug targets and drug resistance mechanisms, which have major implications for fighting malaria. We present a new method for introducing drug resistance mutations in *P*. *falciparum* without the use of plasmids or the need for cloning homologous recombination templates. We demonstrate this method by introducing edits into the sodium efflux channel PfATP4 by transfection of a purified CRISPR/Cas9-guide RNA ribonucleoprotein complex and a 200-nucleotide single-stranded oligodeoxynucleotide (ssODN) repair template. Analysis of whole genome sequencing data with the variant-finding program MinorityReport confirmed that only the intended edits were made, and growth inhibition assays confirmed that these mutations confer resistance to the antimalarial SJ733. The method described here is ideally suited for the introduction of mutations that confer a fitness advantage under selection conditions, and the novel finding that an ssODN can function as a repair template in *P*. *falciparum* could greatly simplify future editing attempts regardless of the nuclease used or the delivery method.

## Introduction

Drug resistance in the deadly malaria parasite *Plasmodium falciparum* is a global problem that continues to plague healthcare efforts even as new drugs are developed and deployed. Drug selections on parasites grown in culture can lead to discovery of resistance-associated genome mutations and shed light on mechanisms of action [1–5]. A necessary complement to this approach is the ability to make targeted mutations in a clean background to assess their impact on drug resistance. Genetic manipulation of *P*. *falciparum* has long been a challenge, but recently a number of groups have developed plasmid-based methods using CRISPR/Cas9 (clustered regularly interspaced short palindromic repeats/CRISPR-associated protein 9) to make insertions and point mutations [6–11]. In some cases CRISPR/Cas9 was used for validating drug resistance mutations [9,12,13]. Zinc finger nucleases have also been used for this purpose [14–17]. Here we describe an alternative method using recombinant Cas9 protein complexed with synthetic guide RNAs (ribonucleoprotein, RNP) and single-stranded oligodeoxynucleotide (ssODN) repair templates. This method requires no molecular cloning and leaves no genetic scar in the parasite save for the intended point mutation(s). RNP-based CRISPR/Cas9 methods are gaining popularity in human and model organism systems, due in part to ease of use and in part to the relatively fast clearance of the editing machinery which reduces the chance of off-target effects. An additional benefit of these methods is their adaptability to non-model organisms for which standard genetic tools are absent or limited.

Previous CRISPR/Cas9 editing methods developed for *P*. *falciparum* use transfected plasmids to introduce all three components of the editing reaction: the Cas9 protein, guide RNAs, and homologous recombination template. Since *P*. *falciparum* parasites lack the machinery to repair DNA double stranded breaks by non-homologous end joining (NHEJ) [18–21], a template is thought to be mandatory for editing, and typically a circular plasmid has been used. The use of such plasmids comes with the risks of unwanted insertion into the genome via single-crossover events [22].

As a test case for RNP-based editing in *P*. *falciparum*, we chose the sodium efflux channel *Plasmodium falciparum* ATPase 4 (PfATP4). This protein is known to be the target of both NITD609 (Novartis) and SJ733, antimalarial compounds currently undergoing clinical trials [23,24]. A number of point mutations in PfATP4 have been discovered over the course of several resistance selection experiments and subsequently characterized [1]. We recapitulate drug resistance by making two single edits in the *pfatp4* gene, each of which confers a greater than 100-fold increase in EC_50_, validating the Cas9 RNP technique and confirming that these mutations in PfATP4 are determinants of resistance.

## Materials and methods

### Preparation of CRISPR/Cas9

The CRISPR/Cas9 protein was prepared in-house as described in [25], except that a C-terminal mRuby2 tag was used instead of mCherry. The construct thus consisted of the following components, listed N terminus to C terminus: 6X HIS tag, maltose binding protein (MBP, to improve solubility), *S*. *pyogenes* Cas9, 2X SV40 nuclear localization site (NLS), mRuby2, 1X SV40 NLS. Briefly, the Cas9 vector was expressed in BL21-CodonPlus (DE3)-RIL competent cells (Agilent, Santa Clara, CA, USA) overnight at 16°C, after which cultures were centrifuged and frozen. Thawed cell pellets were later resuspended in lysis buffer (50 mM sodium phosphate pH 6.5, 350 mM NaCl, 10% glycerol, 1 mM TCEP) supplemented with protease inhibitors and microfluidized. The soluble fraction was purified on a heparin column on the GE ÄKTA pure system, then concentrated down and further fractionated by size exclusion chromatography. The resulting pooled fractions were concentrated and stored at -70°C in 50% glycerol.

### Guide RNA and ssODN template design

To produce dual guide RNAs (dgRNAs), we obtained synthetic trans-activating CRISPR RNA (tracrRNA) as well as CRISPR RNAs (crRNAs) with the following target sequences from IDT (Integrated DNA Technologies, Coralville, IA, USA): L350H: 5`- TTATTTAATGCTACTTGAAG-3`; P412T: 5`-AAGGTGATAGTAACAACCAT-3`.

Two hundred nucleotide single-stranded oligonucleotide homologous repair templates (ssODNs) were designed to be homologous to the wild type ACP strain *pfatp4* save for the desired drug resistance-conferring mutation and 3–7 synonymous marker mutations. The sequences were as follows, with the mutations highlighted in bold text: L350H: 5`- CATCTGTAACTAGCGGTTCTGGTAAAGGTATTGTTATATCCACAGGTTTAGATACACAAGTAGGAAAAATTGCATC**G**CA**G**TTAAAAAAAAGCAGTAAAGGAAGTAAATTAACACCTC**AC**CA**G**GT**T**GC**TC**TAAATAAATTAGGTGGTTTAATTGGTTTAATAGCTATTATTGTATTAGTTGTTATTATCAGCTTAGCTGTT-3`. P412T: 5`-ATATAGAGATCCAGCACATGCAGATAAAGATCCAACCTTTGTTATTATTATTATTGGTGTAGGTTTTGCTGTATCTTCCATTCCAGAAGGTTT**GA**C**G**ATGGTTGT**A**ACTATCACCTTATCAGCAGGAGCTAAAGATATGGTTAAAAAAAATGCAAATGTAAGAAAACTACCAGCTGTTGAAACTTTAGGATGCTGTTCAG-3`. These ssODNs were purchased from IDT and dissolved in H_2_O to a final concentration of 100 μM.

### Parasite cultures and clonal populations

We used the D10-derived ACP_leader_-GFP strain [26], hereafter referred to as ACP. Asexual *P*. *falciparum* ACP parasites were grown in human donor red blood cells (RBCs) at 2% haematocrit (percentage volume of RBCs in total volume culture) in RPMI-1640 media with 2 mM L-Glutamine, 25 mM HEPES, 2 g/L sodium bicarbonate, 5 g/L AlbuMAX II Lipid-Rich BSA (Life Technologies, Carlsbad, CA, USA), 0.1 mM hypoxanthine, 50 mg/L gentamicin, and 100 nM pyrimethamine. Cultures were maintained at 37°C, 5% O_2_, and 5% CO_2_. RBCs were collected from human donors under UCSF IRB number 10–02381.

Clonal populations of ACP were generated by growing serially diluted parasites in a 96-well plate for 3 weeks and then selecting wells that contained infected RBCs. During this time, growth media was replaced every 2–4 days and cultures smeared for detection of parasites from day 14 onwards. The clone obtained from this selection and used in this study was designated ACP-B6.

### Transfection

High parasitemia (17% of RBCs infected with parasites), synchronized ring-stage parasite cultures grown in fresh donor red blood cells at 2% hematocrit were transfected in 96-well Lonza Nucleofector plates (Lonza, Basel, Switzerland). Transfection reagents were mixed in the following order to minimize the risk of Cas9 precipitation and RBC lysis: 2.8 μL of 10X Cas9 activity buffer (500 mM Tris pH 8.0, 1 M NaCl, 100 mM MgCl_2_, 10 mM TCEP), 7 μL of 100 μM Cas9-mRuby2, 11.2 μL of 50 μM guide RNA, 175 μL of Nucleofector Solution SE with supplement added (Lonza), 52.5 μL packed RBCs (at 17% parasitemia), 14 μL of 100 μM single stranded template DNA, and 17.5 μL of 200 mM Na_2_ATP pH 7.4 (Sigma). This resulted in a total volume of 280 μL with final concentrations of 2.5 μM Cas9-mRuby2, 2 μM guide RNA, 5 μM of ssODN, and 18.75% RBCs (at 17% parasitemia). Twenty microliters of this mixture were aliquoted into each of 12 wells of the Nucleofector plate. Thus for each edit, a total of 4.5x10^8^ red blood cells containing 7.7x10^7^ parasites were transfected with 600 pmoles Cas9, 480 pmoles guide RNA, and 1200 pmoles ssODN.

Transfection was done on the Lonza 96-well shuttle on pulse setting CM-162. Post-transfection care was carried out similarly to what is described in [27]: the freshly transfected mixture was incubated for 5 minutes at 37°C, brought up to 100 μL with pre-warmed media, and incubated for another 15 minutes at 37°C. It was then transferred out of the Nucleofector plate into a round bottom 96-well plate containing an additional 100 μL pre-warmed culture media. The transfected parasite-infected RBCs were pelleted at 90 g for 2 minutes, resuspended in fresh pre-warmed media, and incubated at 37°C for 2 to 4 hours before being expanded to a 10 mL culture containing 2% fresh RBCs. For two days, the transfected culture was monitored by Giemsa smearing, and expanded as needed to keep parasitemia at a reasonable level. After 48 hours, the media was replaced and 500 nM SJ733 was added. For 3 days after drug introduction, drug media was replaced daily. By day 6 after the transfection, parasites were no longer detectable in the transfected culture, and drug media was subsequently refreshed every two days. Cultures were monitored by regular smearing. On day 14 and again on day 21 after the transfection, 200 μL of 50% freshly drawn donor RBCs were added to replace old and lysing RBCs. Live parasites were first observed in the L350H culture on day 27 post-transfection (1.7% parasitemia) and in the P412T culture on day 34 post-transfection (0.4% parasitemia). These cultures were designated ACP-B6-L350H and ACP-B6-P412T. Portions were harvested for gDNA collection on day 31 (L350H) and day 41 (P412T), and the remainder maintained for growth inhibition assays.

### DNA isolation and Sanger sequencing

Genomic DNA (gDNA) was isolated from ring-stage parasites with 0.1% saponin lysis and phenol-chloroform extraction as described in [28]. A 400 bp segment of the *P*. *falciparum pfatp4* gene containing both the L350H and P412T mutations was amplified via PCR using the extracted gDNA and primers flanking either end (forward primer: 5`- GGTTTAGATACACAAGTAGGA-3`; reverse primer 5`- TCAGTTAATGTACCGGTTTT-3`. Sanger sequencing by Quintara Biosciences (South San Francisco, CA, USA) of the PCR product using the reverse primer confirmed CRISPR-derived mutations.

### Whole genome sequencing

Paired-end NGS libraries were constructed from ACP-B6, ACP-B6-L350H and ACP-B6-P412T gDNA using the NEBNext Ultra II kit (New England Biolabs, Ipswitch, MA, USA). Two hundred nanograms of gDNA were enzymatically fragmented (with no additional MgCl_2_ supplemented) for 17.5 minutes at 37°C, then the reaction was quenched with EDTA. The samples were cleaned with AMPure beads (Beckman Coulter, Brea, CA, USA) at a sample:bead ratio of 1:1.4 and eluted in sterile H_2_O. Eluted samples were run on a Bioanalyzer HS DNA kit (Agilent, Santa Clara, CA, USA) to confirm the presence of gDNA fragments between 200 and 300 bp long. Samples were then subjected to End Prep as described in the NEBNext Ultra II protocol. Adaptor ligation was performed as described in the same protocol, using a 1:10 dilution of the NEBNext Adaptor. After digestion with USER enzyme, a second AMPure bead clean-up was carried out with a sample:bead ratio of 1:0.9 and an increased incubation time of 15 minutes prior to initially placing the beads on the magnet. Samples were eluted with sterile H_2_O. Using NEB Q5 polymerase, samples were indexed via PCR with unique TruSeq i5/i7 barcode primers. Nine cycles of PCR were conducted according to the NEB Q5 protocol. A final AMPure bead clean-up step was performed at a sample:bead ratio of 1:0.9 and eluted with sterile H_2_O. Library quality was assessed with a Bioanalyzer HS DNA kit and broad peaks from 200 to 500 bp were observed in each sample, as expected. Libraries were pooled and sequenced on an Illumina Hi-Seq 4000 using a PE125 flow cell (Illumina, San Diego, CA, USA).

### Data analysis

Raw data have been deposited in NCBI’s Sequence Read Archive (SRA) under BioProject ID PRJNA360625. (This represents the minimal underlying data set for this study.) After demultiplexing, fastq files were filtered with PriceSeqFilter [29] with the flags “-pair both -rqf 100 .98 -rnf 100,” specifying that in all read pairs 100% of nucleotides must have a 98% chance of being correct, and that no read pairs may contain any ambiguous characters. This resulted in retention of more than half of the reads: for the parent ACP-B6, 5,528,674 read pairs passed filter (57.1% of total reads); for the mutant ACP-B6-L350H, 8,239,818 read pairs passed filter (54.6% of reads); and for the mutant ACP-B6-P412T, 3,974,875 read pairs passed filter (56.5% of total reads).

The filtered fastq files were then aligned to the 3D7 genome from PlasmoDB, version 26 [30], using gmap/gsnap (Genomic Short-read Nucleotide Alignment Program) [31] with the following flags: “—gmap-mode = none—batch = 4—npaths = 1—maxsearch = 10.” The resulting SAM files were imported into Geneious version 8.1.8 [32] and run through MinorityReport to assess genetic differences [33]. Aligned sequencing reads for ACP-B6 and each mutant strain were input along with the FASTA and GFF files for the 3D7 reference genome from PlasmoDB, version 26 [30]. The thresholding flags “-wtc 5,” “-vp 0.6,” and “-vc 5” were used, requiring each reported variant to have a coverage of at least 5 reads in both the parent and the mutant, and at least 60% of the mutant reads to contain the variant.

### Growth inhibition assays

Synchronized ring stage ACP-B6, ACP-B6-L350H and ACP-B6-P412T parasites at 0.8% parasitemia were grown at 0.5% hematocrit in 96-well plates with culture media containing serially diluted SJ733 with concentrations ranging from 3.16 nM to 100 μM, plus a no drug control. All wells contained a final concentration of 1% DMSO; due to the limited solubility of SJ733 this was necessary for achieving drug concentrations sufficient to kill the mutant strains. After 72 hours (1.5 cycles of parasite growth), the trophozoite-stage parasites were fixed with 1% PFA and stained with 50 nM YOYO-1 (Life Technologies). Flow cytometry on an LSRII (Beckman Coulter) was used to determine the percentage of viable parasites. Data were analyzed with FlowJo (Ashland, OR, USA) and dose-response curves were created using 4-parameter non-linear regression with Prism 7 (Graphpad, La Jolla, CA, USA).

## Results

### Design and preparation of CRISPR reagents

**Fig 1:**
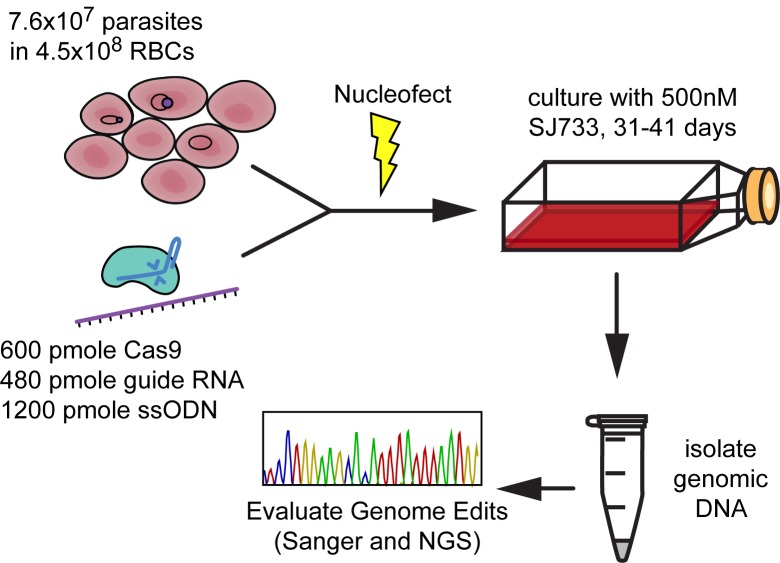
Strategy for introducing plasmid-free CRISPR/Cas9 edits to the *Plasmodium falciparum* gene *pfatp4*. Synchronized ring-stage parasites at 17% parasitemia in fresh donor RBCs were nucleofected with Cas9 protein, guide RNA, and template ssODN. Cultures were kept under drug pressure with 500 nM SJ733 starting on day two post transfection. After drug-resistant parasites emerged from culture, genomic DNA was isolated with standard phenol-chloroform extraction methods for library preparation. The presence and penetrance of the targeted CRISPR edits were confirmed using Sanger sequencing and whole genome NGS.

**Fig 2:**
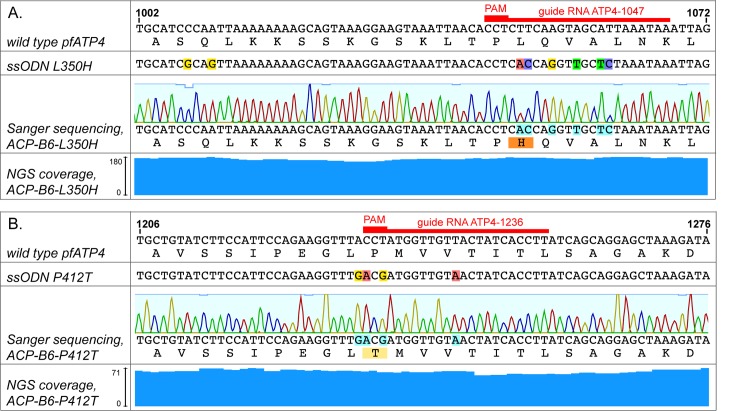
Sequencing results. Sanger and NGS sequencing coverage of targeted CRISPR mutations at the *pfatp4* locus for ACP-B6-L350H and ACP-B6-P412T with clonal wild type parent strain ACP-B6. Red bars delineate the respective 20 nt guide RNA target sites and PAM sites required for each edit. NGS coverage at each location is indicated by blue columns. (**a**) Sequencing data of targeted locus 1002–1072 in *pfatp4* from strain ACP-B6-L350H showing SJ733 resistance-conferring SNPs in L350 and four other synonymous mutations introduced by CRISPR. Sequences of wild type *pfatp4* and repair template ssODN L350H are shown in alignment. The two silent mutations in ssODN L350H located 39 and 42 nt away were not incorporated into ACP-B6-L350H. (**b**) Sequencing data of targeted locus 1206–1276 in *pfatp4* from strain ACP-B6-P412T showing the SJ733 resistance-conferring SNP and silent mutations introduced by CRISPR. Sequences of wild type *pfatp4* and repair template ssODN P412T are shown in alignment.

Fig 1 illustrates the simple design of this study. The sodium efflux channel PfATP4 was chosen as an editing target because of its multiple known mutations, derived by growth under drug pressure, that putatively confer a high degree of resistance to the novel dihydroisoquinolone antimalarial compound SJ733. These include, among others, L350H and P412T [1]. When designing guide RNAs to target these sites, we selected the PAM sites closest to each desired mutation location. Guide RNA PfATP4-1047 recognizes the PAM site 5`-CCT-3`and the target 5`-CACCAGGTTGCTCTAAATAA-3`at position 1045–1057 and cuts between nucleotides 1050 and 1051. Guide RNA PfATP4-1236 recognizes the PAM site 5`-CCT-3`and the target 5`-ATGGTTGTTACTATCACCTT-3`at position 1234–1256 and cuts between nucleotides 1239 and 1240 (see Fig 2).

For guide RNA PfATP4-1047, the 200 nt ssODN repair template includes the two nucleotide changes that make up the L350H mutation plus additional silent mutations that would allow us to confirm generation of a CRISPR-directed mutation rather than a spontaneous drug resistance mutation. Since the PAM site for this guide RNA cannot be ablated without making a non-synonymous mutation, we included four silent mutations in the target region adjacent to the PAM site, for a total of six mutations, to decrease the chance of re-cutting after the ssODN has been incorporated into the genome (see Fig 2A). This ssODN also has two additional mutations located 39 and 42 nt away from the cut site. These are one PAM-ablating and one confirmatory silent mutation, added to allow future use of this ssODN with an alternative guide RNA target site.

For guide RNA PfATP4-1236, we designed a 200 nt ssODN repair template that included the P412T mutation and three additional confirmatory silent mutations. The P412T mutation also ablates the PAM site, meaning that once an edit has occurred in the parasite, the guide RNA PfATP4-1236-directed Cas9 RNP would not be able to cut it again (see Fig 2B).

Cas9 protein tagged with mRuby2 was recombinantly expressed in *E*. *coli*. Synthetic crRNAs, tracrRNA, and ssODNs were commercially synthesized.

### Transfection of parasites with CRISPR reagents

We used a *P*. *falciparum* strain designated ACP-B6, which was cloned by limiting dilutions from the D10-derived ACP_leader_-GFP strain [26]. We synchronized the parasites and grew them to a high density. At the time of transfection, the culture was at 17% parasitemia, of which 84% were ring stage.

Cas9 protein, guide RNAs and ssODNs were mixed with the parasite culture in appropriate buffer conditions, as described in detail in the methods section. The mix was added to a Lonza Nucleofector 96-well plate, 20 μL per well in a total of 12 wells for each mutation. The pulse setting used was CM-162; a setting that our group previously determined to be optimal for transfection into RBCs [27].

After 48 hours of drug-free culturing following the transfection, cultures were maintained in media supplemented with 500 nM SJ733 and monitored by regular Giemsa smearing. Parasites were undetectable at day 6 after transfection, and live parasites re-emerged in the L350H culture on day 27 (1.7% parasitemia) and in the P412T culture on day 34 (0.4% parasitemia). Cultures were harvested for gDNA collection on day 31 and day 40, respectively.

### Sequencing confirms successful introduction of mutations

#### Sanger sequencing

Fragments of *pfatp4* containing the edited sites were PCR-amplified from gDNA and Sanger sequenced. As depicted in Fig 2, the sequences surrounding the cut sites were identical to that of the ssODNs, with the resistance-causing mutations and all the adjacent silent mutations present. Interestingly, the two additional mutations in the L350H ssODN located 39 and 42 nt away from the cut site were not incorporated into the *pfatp4* gene, suggesting that a crossover event happened within 39 bp of the cut site.

#### Next generation sequencing

Three Illumina libraries, for the parent clonal culture (ACP-B6) and the two edited cultures (ACP-B6-L350H and ACP-B6-P412T), were prepared from gDNA using the NEBNext Ultra II Library Prep kit and sequenced using a PE125 flow cell on an Illumina HiSeq 4000. Filtered datasets were aligned to the *P*. *falciparum* genome (3D7, PlasmoDB-26) using gsnap [31]. The resulting SAM files were imported into Geneious [32] for analysis of alignments. No read trimming was performed. The ACP-B6, ACP-B6-L350H and ACP-B6-P412T libraries yielded 58-fold, 85-fold and 28-fold coverage of the *P*. *falciparum* genome, and 169-fold, 189-fold and 63-fold average coverage of the *pfatp4* gene, respectively. The parent strain had 189 reads covering the genomic location of L350 and 168 reads covering that of P412, of which 187 (98.9%) and 166 (98.8%), respectively, matched the reference sequence. ACP-B6-L350H had 174 reads covering L350H, of which 173 (99.4%) matched the 6 mutations surrounding the cut site designed into the L350H ssODN. NGS also confirmed that the two additional mutations located 39 and 42 nucleotides away from the L350H cut site in the L350H ssODN were not incorporated into the genome (175 and 179 reads of coverage, of which 173 (98.9%) and 179 (100%) match wild type, respectively). ACP-B6-P412T had 63 reads covering P412T, of which 62 (98.4%) exactly match the mutations designed into the P412T ssODN (Fig 2).

To confirm the mutation analyses and assess the presence of off-target mutations, we applied MinorityReport, a software package developed in our lab [33]. This script takes as input two SAM files, one for the parent (ACP-B6) and one for the mutant (ACP-B6-L350H or ACP-B6-P412T), as well as the FASTA and GFF files of the *P*. *falciparum* genome. It identifies discrepancies (nonsynonymous snps, insertions, deletions, and copy number variation) between the parent and the mutant, and reports them with annotations drawn from the GFF file. This analysis confirmed the presence of the intended L350H edit at high penetrance (97.9%) in ACP-B6-L350H, and of the intended P412T edit at high penetrance (96.5%) in ACP-B6-P412T, and the absence of both mutations in the parental strain. No other mutations or copy number variants were identified by MinorityReport for either strain. Thus, we conclude that no additional drug resistance mutations occurred spontaneously, or by off-target editing in these cultures. The MinorityReport results are presented in S1 Table and S1 Fig.

The absence of off-target effects associated with these CRISPR-induced mutations was expected, since no significant off-target matches to either of these guide RNA sites are present in the genome, as confirmed by running the off-target prediction program EuPaGDT [34] with default settings, using as input sequences the regions surrounding the two guide RNA target sites. To push the limits of the off-target detection, we also ran EuPaGDT with more permissive off-target parameters: seed sequence length (including PAM) of 8 and maximum mismatches of 8. This yielded no off-target sites for guide RNA PfATP4-1236 with an intact NGG PAM site, and only a single site for PfATP4-1047 with an intact NGG PAM site. This site has the target sequence 5`- **AA**A**AG**T**T**ATGCTACTTGAAG-3`(where nucleotides in bold represent the five mismatches relative to guide RNA PfATP4-1047) followed by the PAM site 5`-AGG-3`, and lies within the gene PF3D7_1245500 on chromosome 12. Sixteen NGS reads from ACP-B6-L350H map to this site, all of them with wild type sequence, indicating that no CRISPR edits occurred there (S2 Fig).

#### Drug resistance

**Fig 3:**
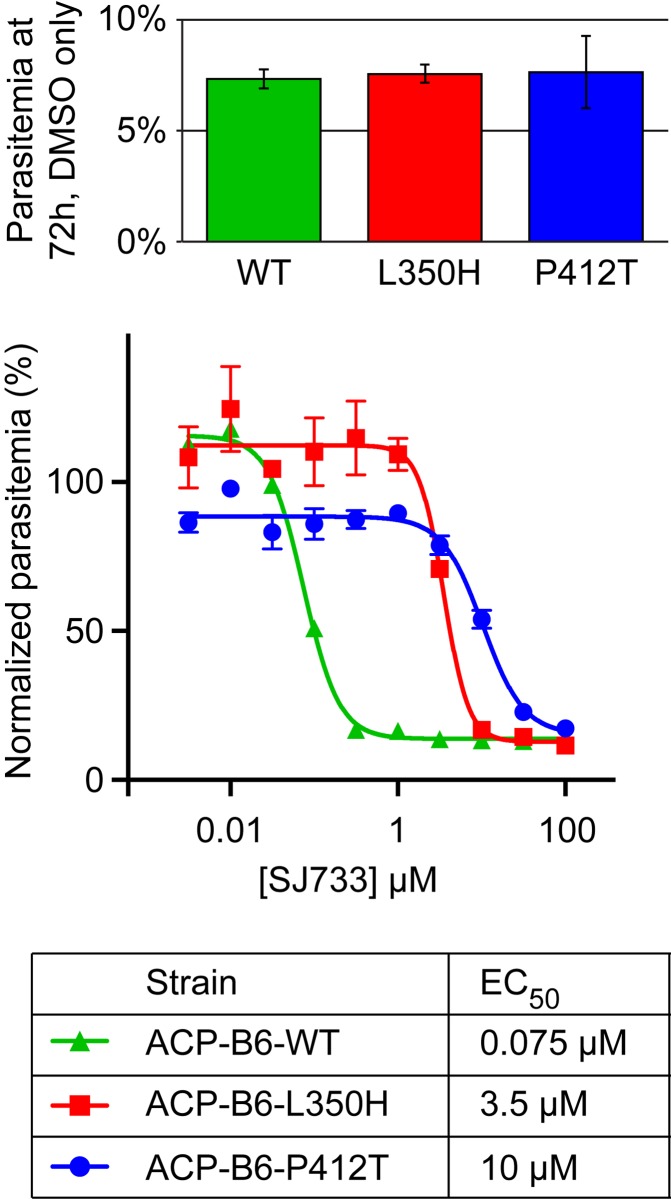
Characterization of drug resistance. Dose-response curves and EC_50_ values for the antimalarial SJ733 on the parent strain ACP-B6 and the mutants ACP-B6-L350H and ACP-B6-P412T. The growth inhibition assay was conducted by seeding synchronized ring-stage parasites from each strain at 0.8% parasitemia in media supplemented with SJ733 at concentrations ranging from 3.16 nM to 100 μM and allowing for growth over 72 hours. Parasites were fixed with 1% paraformaldehyde and stained with 50 nM YOYO-1. Final parasitemia was assessed by flow cytometry and values were normalized to DMSO-only controls. Values reported are mean ± standard error (n = 3). The inset shows parasitemia of each culture after 72 hours of growth in the presence of DMSO only.

To confirm that these CRISPR-induced mutations confer resistance to the drug SJ733, we performed a standard growth inhibition assay. We found SJ733 EC_50_s of 75 nM on wild type ACP-B6 parasites, 3.5 μM on ACP-B6-L350H and 10 μM on ACP-B6-P412T, indicating 47- and 138-fold increases in resistance, respectively (Fig 3). These findings are consistent with previously published data on parasites that spontaneously adopted these mutations while under drug pressure in culture [1] and confirms that these mutations are determinants of resistance.

## Discussion

We have described a plasmid-free method for introducing drug resistance mutations into the malaria parasite *P*. *falciparum* using CRISPR/Cas9. The absence of a need for molecular cloning and the scar-free nature of this technique make this an attractive editing method especially for mutations that confer a natural or drug-based fitness advantage. We validated the method by introducing precise edits into the drug target PfATP4, which recapitulated drug resistance to the clinical candidate compound SJ733.

While the reagents required for this method are fast and easy to generate, we found that no post-transfection time was saved relative to what has been reported for plasmid-based CRISPR editing methods [6–10], most likely due to the low efficiency of transfection of both RNP and plasmids. Indeed, even with the optimized technique described here, only 6 (23%) out of a total of 26 transfections into the ACP strain with both guide RNA PfATP4-1236 and a P412T ssODN resulted in live parasite cultures with successful CRISPR mutations. The remaining 20 transfections failed to yield genome edits. The underlying reasons for this relatively low success rate are likely to be multi-factorial. Plasmodium is a complex organism, and the parasitized red blood cell features multiple membranes that must be breached for any transfection to be successful; indeed, low efficiency of plasmid-based transfections is a problem that has long plagued genetics research in this parasite. Further, there is no evidence that *P*. *falciparum* possesses the capability for non-homologous end-joining (NHEJ) [18–21]–this presents yet another barrier to efficient editing, as not only the CRISPR/Cas9 complex but also the template DNA must be present at the location of editing, and furthermore the template provided must compete with the sister chromatids that would normally function in repair. Future research into these issues may lead to improvements in the efficiency of this and all genome editing methods.

Given the relative complexity of this system, we were encouraged to see that our plasmid-free technique does yield reproducible success. We have demonstrated the first use of ssODNs as repair templates for CRISPR editing in the malaria parasite in the context of co-transfection with the Cas9/sgRNA RNP. The use of ssODNs is particularly attractive given that they are readily commercially available and obviate the necessity for cloning. Further, other CRISPR editing studies have typically used long double stranded repair templates, which are especially time-consuming to generate for this extremely AT-rich organism. These plasmids also come with the risk of multiple copies inserting into the genome due to single crossover events. Therefore, an optimal editing strategy for making point mutations that do not confer drug resistance may be to use selectable, plasmid-based Cas9 and guide RNAs with 200 nucleotide ssODN repair templates.

## Supporting information

S1 Fig MinorityReport CNV image output. (EPS)

S2 Fig Off-target site. NGS confirms no editing of the single possible off-target site of guide RNA PfATP4-1047. This site lies within the gene PF3D7_1245500 on chromosome 12 and is covered by 16 wild type reads from the ACP-B6-L350H sequencing library. Nucleotides in orange represent mismatches relative to guide RNA PfATP4-1047.
(EPS)

S1 Table MinorityReport output. (XLSX)
